# The Boy across the Street

**Published:** 1907-08

**Authors:** Thos. H. Glover

**Affiliations:** San Marcos


### The Boy Across The Street.

BY THOS. H. GLOVER,* OF SAN MARCOS.

We are indebted to Dr. S. W. Cock, of Mineral Wells, Texas, for a copy of the *Mineral Wells Resort* containing this gem. Dr. Cock owns the original. The poem has never been published but once before. It appeared in the *Mirror*, of St. Louis, some ten years ago.—ED.

I jus' wish I was that boy across the street,
Nobody combs his hair and keeps him neat;
He wears a great big, broadbrim, "tored-up" hat,
And plays in the street with his ball and bat,
And goes barefooted.

He can go to the creek to fish and swim,
And nobody 'tall is afraid for him.
Don't wear stiff shirts and stay clean when he plays;
Runs out with a yell and jumps on the drays,
And goes barefooted.

I wish I was that boy across the street, 'cause
Nobody says "you mustn't" to everything he does.
He can whip mos' any boy his size in town,
And take in ever' circus, and holler at the clown,
And goes barefooted.

(Views of the Boy Across the Street.)

Don't I thes wish my dad was rich
Like that sissy boy's across the way?
I'd pitch this ol' hat in the ditch,
And have pie for dinner ever' day.
If I was him.

I'd wear the best of shoes, of course,
So soles and uppers wouldn't part;
And drive a spotted pony horse
To a bran'-new, yaller-painted cart.
If I was him.

I'd throw away this crooked lim',
And buy me a nice, long, j'inted pole,
With a reel and line what's strong, but slim,
Then ever' fish I hooked I'd hol',
If I was him.

I needn't crawl in under the tent
Whenever a circus comes to town,
'Cause all my money wouldn't be spent,
And I'd buy a song book from the clown,
If I was him.

---

*Glover was a genius. Like Poe, Keats, Chatterton, and others of his kind, he died young (Died December 8, 1902.)

